# Interplay between polycystic ovary syndrome and hypothyroidism on serum testosterone, oxidative stress and *StAR* gene expression in female rats

**DOI:** 10.1002/edm2.359

**Published:** 2022-07-24

**Authors:** Sara Khodabandeh, Abdolkarim Hosseini, Homayoun Khazali, Vahid Azizi

**Affiliations:** ^1^ Faculty of Life Sciences and Biotechnology Shahid Beheshti University Tehran Iran

**Keywords:** hypothyroidism, polycystic ovary syndrome, steroidogenic acute regulatory protein, testosterone

## Abstract

**Introduction:**

Endocrine disorders such as polycystic ovary syndrome (PCOS) and hypothyroidism can cause infertility. There are evidence that they happen jointly in some circumstances. It still remains unknown, how these two illnesses interact and influence the body.

**Methods:**

Accordingly, a five‐group was designed, first is the control group, followed by the PCOS group. Estradiol valerate (EV) induced PCOS, the second group had only PCOS and the third, fourth and fifth groups were given varied dosages of propylthiouracil (PTU) to cause hypothyroidism after induction of PCOS. Steroidogenic acute regulatory protein (StAR) expression was measured in the ovaries, and serum was obtained to determine testosterone levels, as well as superoxide dismutase (SOD) as an antioxidant and malondialdehyde (MDA) as an oxidant.

**Results:**

Based on radioimmunoassay data, testosterone levels were significantly higher in the PCOS group than the control group, and significantly lower (*p* ˂ .05) in PTU groups comparing with the PCOS group. According to the quantitative real‐time polymerase chain reaction (qRT‐PCR) data, the same results were obtained for the *StAR* gene as well. The data also indicated a positive correlation between these two. Although both oxidant and antioxidant level increased in PCOS group compared than control group, after hypothyroidism, oxidant level increased significantly (*p* ˂ .05), meanwhile antioxidant level decreased significantly (*p* ˂ .05).

**Conclusions:**

The results of this study illustrate that the presence of both PCOS and hypothyroidism alters the situation more than just PCOS. They also indicate that this situation is associated with imbalanced oxidative/antioxidative status.

## Graphical abstract

Based on radioimmunoassay data, testosterone levels were significantly higher in the PCOS group than in the control group, and significantly lower (*p* < .05) in PTU groups than the PCOS group. According to the quantitative real‐time polymerase chain reaction (qRT‐PCR) data, the same results were obtained for the StAR gene as well. The data also indicated a positive correlation between these two. Although both oxidant and antioxidant level increased in PCOS group compared with control group, but after hypothyroidism, oxidant level increased significantly (*p* < .05), but antioxidant level decreased significantly (*p* < .05).

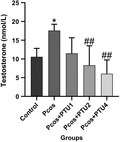

## 1 INTRODUCTION

The most prevalent cause of prolonged anovulation and infertility is the polycystic ovary syndrome (PCOS). PCOS is an endocrine condition that affects women of reproductive age and is linked to metabolic disorders as well as reproductive dysfunction. Because of an imbalance of women's sex hormones, cysts occur in the antral follicles of the ovary in this disorder. The transformation of an egg into a cyst, known as a functional cyst, prevents ovulation. Anovulation causes amenorrhea or the termination of the sexual cycle.^1^

Untreated or undetected thyroid disease can also cause infertility. Thyroid dysfunction can influence fertility by causing irregular cycles, luteal phase deficit, high prolactin levels and sex hormone imbalances.^2^ Thyroid dysfunctions are the most common endocrine problems in the world. Among different thyroid dysfunctions, prevalence of hypothyroidism is about 4%–5% more across the world. Women are in greater danger than men.^3^

Subclinical hypothyroidism (SCH) can exist in women with PCOS. In this subject, different studies have produced diverse outcomes. Some studies suggest that there is a link between these two disorders,^4^ whereas others have found no conclusive evidence.^5^ It should be emphasized that a diagnosis of SCH does not rule out the possibility of PCOS and having both diseases should be considered.

Despite the fact that the definition of PCOS has evolved over time, all diagnostic criteria contain two or more of the following symptoms: oligomenorrhea, oligoovulation/anovulation, androgen excess, as well as polycystic ovaries.^6^ Hyperandrogenism is currently described in two ways: clinically as hirsutism with a Ferriman–Gallwey score of 8 or biochemically as high total or free testosterone. So, measuring testosterone can be a way to diagnose PCOS.^7^

Kahsar‐Miller et al.,^8^ observed alterations in *StAR* expression in 2001, and they suggested that these changes could be linked to reproductive diseases such as PCOS. The *StAR* gene was named in 2005 as one of the genes associated in PCOS. The *StAR* gene produces StAR protein, which is a crucial enzyme in the production of steroid hormones such as testosterone.^9^

By disturbing the ratio of pro‐oxidant molecules such as reactive oxygen species (ROS) and reactive nitrogen species (RNS) and defensive antioxidants, oxidative stress appears to play a key role in the pathophysiology of infertility in both men and women.^10^ When this balance is disrupted, cells and reproductive tissues become unstable. There is substantial evidence that an increase in inflammatory factors is linked to an increase in ROS generation, that the two are intimately linked and, as a result of their interactions, contribute to tissue damage.^11^ Unstable radicals are extremely reactive and obtain their electrons from intracellular biological components such as lipids, proteins, nucleic acids and carbohydrates, causing cell damage.^12^

Androgen biosynthesis is regulated by two cytochrome P450 enzymes: P450 SCC, which catalyses cholesterol side‐chain cleavage, and P450 c17, which catalyses 17‐hydroxylation and 17–20 bond cleavage (17–20 lyase) necessary for dehydroepiandrosterone (DHEA) and a formation from pregnenolone and progesterone, respectively. All steroid synthesis is slowed by the side‐chain cleavage enzyme and the steroidogenic acute regulatory protein (StAR).^13^

Since 1993, when research on hypothyroidism and PCOS at the same time began, the results have been contradictory, and some elements of the condition remain unclear.^14^ This emphasizes the importance of this study. As a result, the goal of this study was to uncover some details of these two disorders.

## 2 MATERIAL AND METHODS

### 2.1 Animals

Twenty five female Wistar rats weighing about 170–180 g and at the age of approximately 6 weeks were kept at a temperature of 22 ± 2°C and a humidity of 50 ± 5 for a week of acclimatization. These animals were chosen randomly. It is worth noting that rats were examined for abnormalities and those found to be abnormal were excluded from further study. The rodents were maintained at alternating periods of 12 h of light and 12 h of darkness. Additionally, there were not any limitations for food or water. The research design was approved by the Shahid Beheshti University Research Ethics Committees, Tehran, Iran (Approval ID: IR.SBU.REC.1400.061).

### 2.2 Material

Estradiol valerate (EV; Aburaihan Pharmacy Co.) and propylthiouracil (PTU; Iranian Hormone Pharmacy Co.) were employed in this experiment. The testosterone hormone was measured using a testosterone assay kit (Izotop Co.), and a total RNA extraction kit (Parstous Co.) was used to extract RNA, followed by a cDNA synthesis kit (Parstous Co.) to synthesize cDNA based on RNA, and finally, a real‐time PCR kit (BioFACT) to evaluate relative gene expression. Malondialdehyde (MDA) and superoxide dismutase (SOD; Navand Salamat Co.) assay kits were used to measure oxidant and antioxidant, respectively. SOD was measured at 405 nm, while MDA was measured at 550 nm.

### 2.3 General procedure

These rats were allocated into five groups at random (Simple Random Sampling; *n* = 5; Table 1). At first, EV was used to cause PCOS, it was administered intramuscularly once during the estrus phase with 2 mg of EV per kg of body weight diluted in olive oil.^15^ The vaginal smear was used to determine the estrus phase. After that, animals were allowed approximately 60 days to fully induce PCOS in them, which were identified via a vaginal smear. The animals of 3 groups (groups 3, 4 and 5) were given oral PTU proportional to their weight for 7 days after this period.^16^

**TABLE 1:** Groups and dosage of EV and PTU

Groups	EV (mg/kg)	PTU (mg/kg)
Group 1 (Control)	‐	‐
Group 2 (PCOS)	2	‐
Group 3 (PCOS+PTU1)	2	1
Group 4 (PCOS+PTU2)	2	2
Group 5 (PCOS+PTU4)	2	4

Abbreviations: EV, estradiol valerate; PTU, propylthiouracil.

### 2.4 Serum collection and its related analysis

Blood samples were taken and using a centrifuge, serum samples were obtained. At the end of the treatment period and after the animals had been anaesthetised with a combination of ketamine (Alfasan co.; 80 mg/kg) and xylazine (Alfasan co.; 10 mg/kg), then the serums were stored in a freezer at a temperature of −20°C. Testosterone assays (nmol/L) were performed by radioimmunoassay according to the kits' procedure (sensitivity, intra‐ and inter‐assay coefficients of variation of the kit were 0.99 nmol/L, 7.3% and 12%, respectively). According to the kits' protocols (Navand Salamat Co.), serum samples were also utilized to assess SOD (U/ml) by autooxidation of Pyrogallol and MDA (nmol/ml) by colorimetric method.

### 2.5 Ovarian preparation and real‐time quantitative polymerase chain reaction

Surgical methods were used to retrieve both ovaries from each rat. These ovaries were then stored at −80°C until more research could be conducted. Total RNA was collected from the experimental groups' ovaries using a total RNA extraction kit (Parstous Co.). Each gene's specific primers were used to measure the target genes (Table 2). Oligo7 software was used to create primers based on their gene sequences. The amount of total RNA was measured by multi‐mode reader. The extracted RNA (1 μg) was then used to make cDNA with the help of the EasyTM cDNA Synthesis Kit (Parstous Co). All of the preceding stages were performed in accordance with the manufacturer's instructions.

**TABLE 2:** Gene ID, forward and reverse primer sequences for the associated genes evaluated by RT‐qPCR in the current study are listed below

Gene name	Gene Bank accession number	Primer sequence (5´→3´)
*StAR*	NM_031558	F‐TGTACCAAGCGTAGAGGTTC
		R‐GCATCTCCCCAAAGTGTG
*Gapdh*	NM_017008	F‐AACGACCCCTTGATTGACCT
		R‐GGTTTCCCGTTGATGACCAG

With gene's particular primer, RT‐qPCR was performed to measure the target gene. The following heat cycle was used to execute PCR in duplicate with a final volume of 20 μl: 10 min at 95°C; 3 cycles of 30 s at 95°C, 30 s at 60°C and 30 s at 72°C. The cycle threshold (Ct) for each reaction was calculated based on efficiency to correct the transcript level analysis. After that, ∆Ct was computed by subtracting the calibrator's average from the Ct of each gene. Finally, using the formula 2^−∆∆Ct^,^17^ the relative expression of each target gene was computed in comparison with the reference gene (*Gapdh*). All of the above steps were completed according to the manufacturer's guidelines.

### 2.6 Statistical analysis

The results were analysed using Graph Pad Prism 8 software. The Shapiro–Wilk test was used to ensure that the data was normal. If the total *F* value was statistically significant during statistical tests such as one‐way ANOVA analysis, Tukey's post hoc test was used to compare groups. In addition, a Pearson test was used to determine whether there was a correlation. Data were presented as mean ± standard deviation (SD), all *p* values were considered significant at *p* ˂ .05.

## 3 RESULTS

### 3.1 Mean serum testosterone levels

**FIGURE 1:**
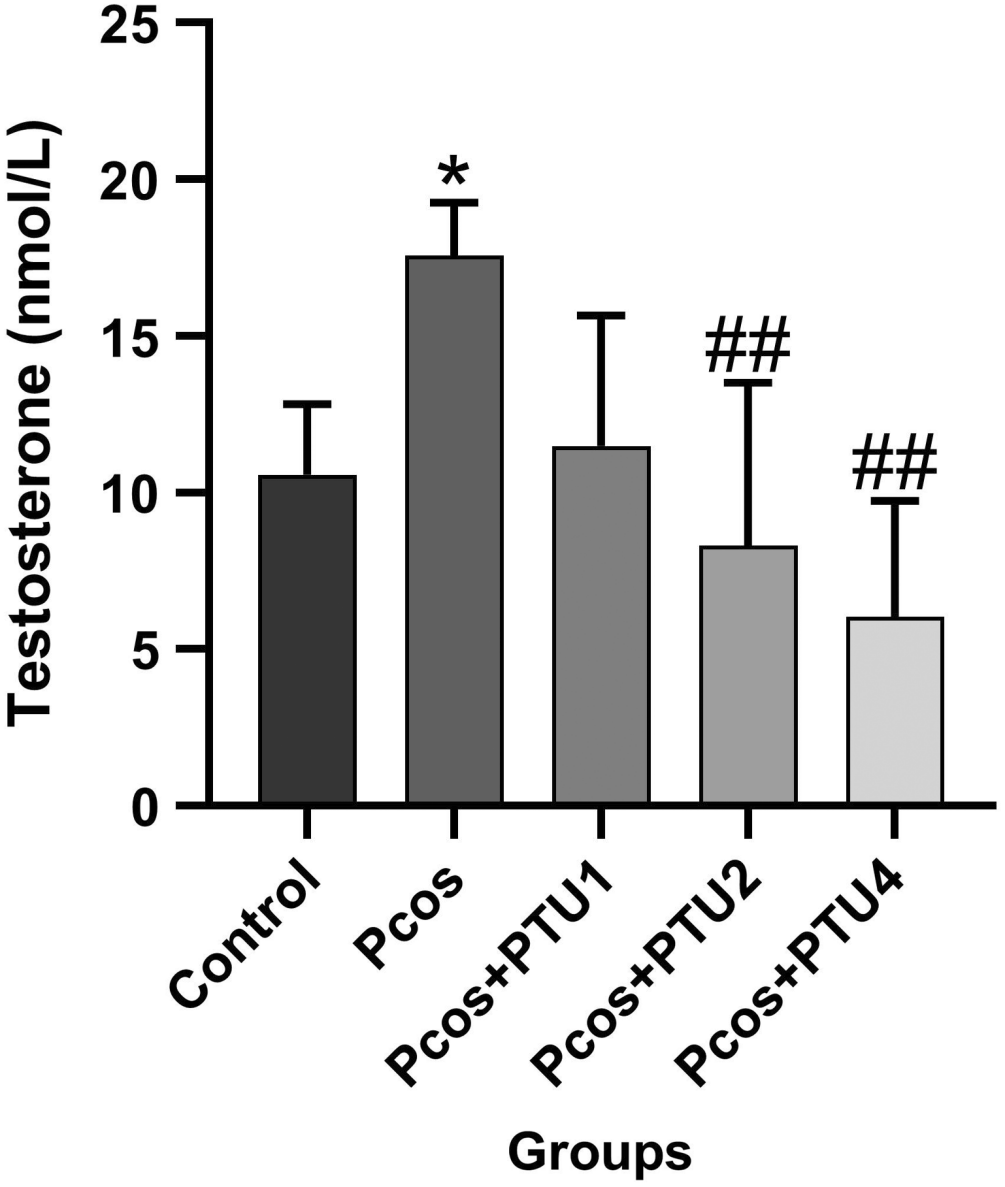
Mean serum testosterone levels (nmol/L) after treatment with PTU to induce hypothyroidism (groups 3, 4 and 5) in rats with PCOS compared with control group (group 1) and PCOS group (group 2; *n* = 5 in each group). Result from each group is represented as mean ± SD. * *p* ˂ .05 versus group 1; ## *p* ˂ .01 versus group 2. PCOS, polycystic ovarian syndrome; PTU, 6‐n‐propylthiouracil

In diverse groups, testosterone levels got varied. A one‐way ANOVA revealed that there was a statistically significant difference in testosterone levels between at least two groups [*F*(4, 20) = 7.098, *p* ˂ .001]. The PCOS group that received EV exhibited a significant increase (*p* ˂ .05, *p* = .04) in comparison with the control group that only received olive oil. Furthermore, the data show that groups 4 and 5, who received 2 and 4 mg/kg of PTU, respectively, had lower testosterone levels than the PCOS group (*p* ˂ .05; *p* = .008 and *p* = .001, respectively; Figure 1).

### 3.2 Relative expression of *StAR* gene

**FIGURE 2:**
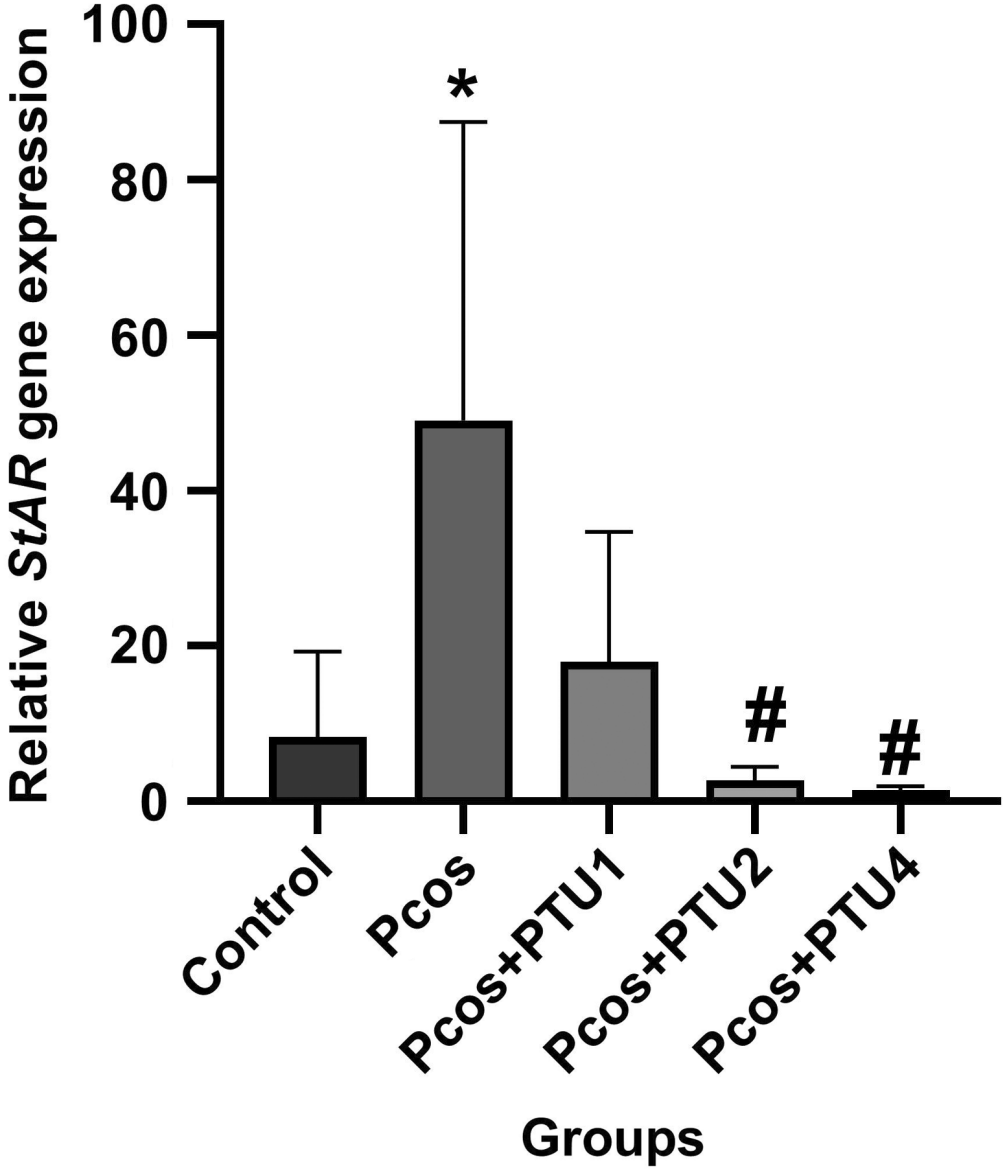
Relative expression of *StAR* mRNA in the rat ovaries after treatment with PTU to induce hypothyroidism (groups 3, 4 and 5) in rats with PCOS compared with control group (group 1) and PCOS group (group 2; *n* = 5 in each group). Result from each group is represented as mean ± SD. * *p* ˂ .05 versus group 1; # *p* ˂ .05 versus group 2. PCOS, polycystic ovarian syndrome; PTU, 6‐n‐propylthiouracil

A one‐way ANOVA revealed that there was a statistically significant difference in StAR gene expression between at least two groups [*F*(4, 20) = 4.149, *p* ˂ .001]. In the PCOS group (group 2), relative expression of the *StAR* gene in the ovaries was significantly higher (*p* ˂ .05, *p* = .03) than the control group. There was a decline in groups 3, 4 and 5 which received PTU and EV. However, only groups 4 and 5 show a significant decrease (*p* ˂ .05, *p* = .04 and *p* = .02, respectively; Figure 2).

### 3.3 The correlation between testosterone and *StAR* gene

**FIGURE 3:**
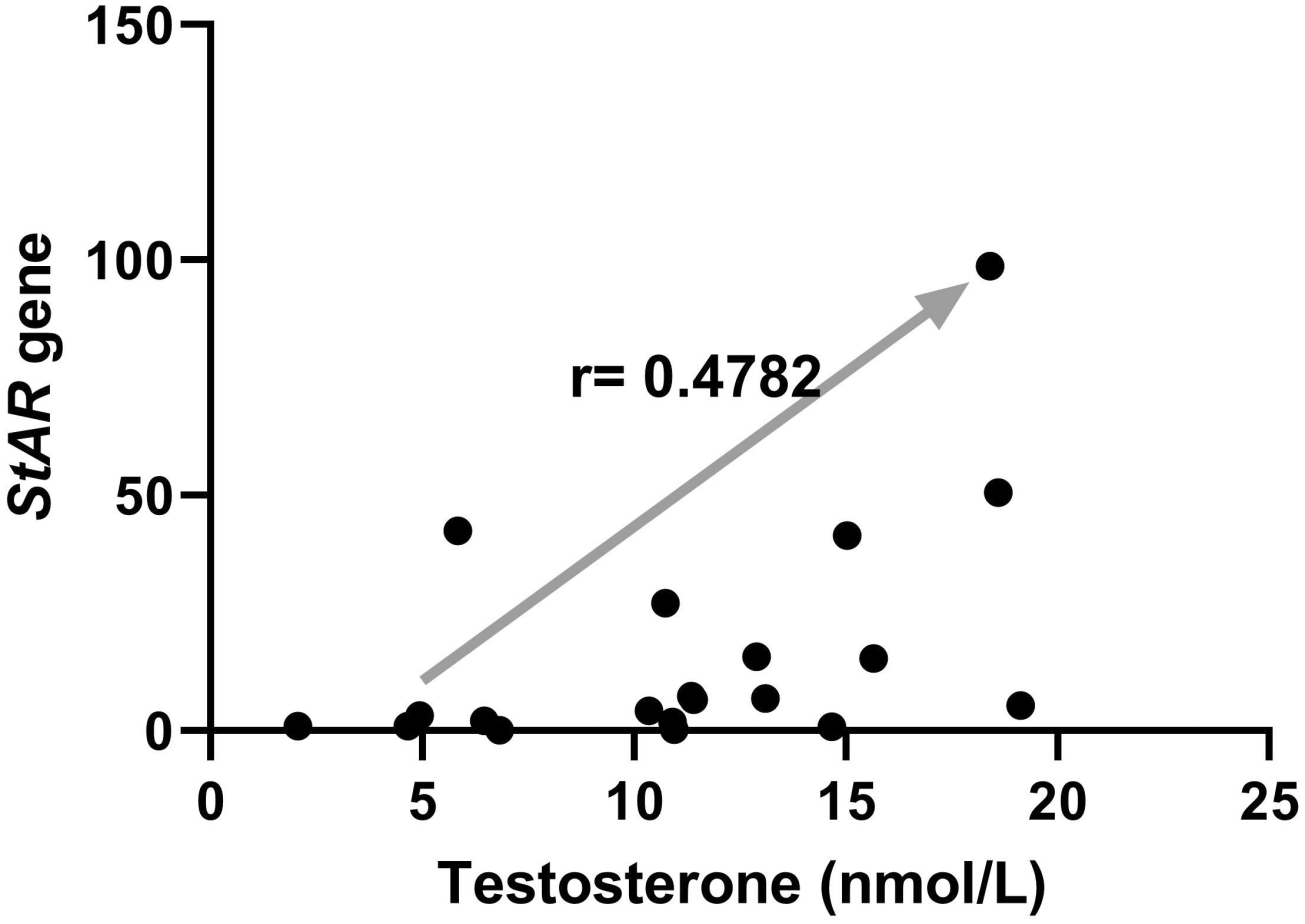
Correlation between testosterone and *StAR* gene. This correlation is reported as *r* = 0.4782 which *r* > 0 means a positive correlation

During this study, the results showed a significant association. These two variables have a positive correlation. As a result, as testosterone levels rise, so does the expression of the *StAR* gene, and vice versa (Figure 3).

### 3.4 Changes in oxidant and antioxidant levels

Two different one‐way ANOVA revealed that there was a statistically significant difference in SOD levels between at least two groups [*F*(4, 20) = 6.720, *p* ˂ .001] and a statistically significant difference in MDA levels between at least two groups [*F*(4, 20) = 7.448, *p* ˂ .001]. When comparing the PCOS group to the control group, SOD levels increased significantly (*p* ˂ .05, *p* = .0019). MDA showed a rise as an oxidant as well; however, it was not significant. SOD levels declined in a dose‐dependent manner in groups that received PTU in addition to EV, but only in the group that received the most PTU did they notice a significant drop (*p* ˂ .05, *p* = .0025). MDA levels, on the contrary, show growth after PTU treatment. Only the group that received 4 mg/kg of PTU experienced significant increases in comparison with control group and also PCOS group (*p* ˂ .05, *p* = .0024, *p* = .0047; Table 3).

**TABLE 3:** Level of SOD and MDA in different groups

Experimental groups	SOD (U/ml)	MDA (nmol/ml)
Group 1 (Control)	384.3 ± 70.29	11,341 ± 1742
Group 2 (PCOS)	935.5 ± 169.1*	19,671 ± 2601
Group 3 (PCOS+PTU1)	661.9 ± 362.2	24,824 ± 6408
Group 4 (PCOS+PTU2)	589.1 ± 120.5	95,435 ± 59,059
Group 5 (PCOS+PTU4)	400.1 ± 96.82#	138,965 ± 84,392*,#

*Note*: Data are represented as mean ± SD (*n* = 5 in each group). * and # shows the significant level at *p* ˂ .05 (*vs. group 1 and # vs. group 2).

Abbreviations: MDA, malondialdehyde; SOD, superoxide dismutase.

## 4 DISCUSSION

What stands out of the results is that while *StAR* gene expression and, as a result, testosterone levels increased in the PCOS group comparing with the control group, they decreased in a dose‐dependent manner after hypothyroidism, with this increase being significant in the PCOS group compared with the control group, and those decreases were also significant in the groups that received 2 and 4 mg/kg of PTU compared with the PCOS group. As predicted by statistics, there is a positive relationship between the *StAR* gene and testosterone levels. In the PCOS group, statics revealed an increase in oxidant and antioxidant levels as compared with the control group, however, only the SOD level was significantly higher. Although SOD levels declined after PTU consumption, MDA levels increased in a dose‐dependent pattern. However, when compared with the PCOS group, these drops and surges were only significant in the group that got the most PTU.

In PCOS, the primary disorder is a large amount of androgen production by the ovaries, which activates a large number of follicles and as a result, the normal amount of FSH is not responsive. Thus, a dominant follicle will rarely be produced.^18^ Various studies in both animal^19^ and human^20^ cases reveal that after getting PCOS, testosterone levels rise because of hormones imbalances. The findings of this study also show that testosterone levels rise following EV‐induced PCOS. The current study also found that the PCOS group had significantly lower testosterone levels than the control group and groups that received 2 and 4 mg/kg of PTU compared with the PCOS group. It also implies that the reduction in the group receiving the most PTU is greater. Another study in this area found that the severity of hypothyroidism is linked to PCOS characteristics, which could be one of the reasons why the lowest dose of PTU failed to produce meaningful results.^14^ The current findings are similar to those of the previous studies on testosterone levels and hirsutism. In that study, testosterone levels were shown to be greater in the PCOS group than in other groups, including the hypothyroid group.^21^

Because the StAR protein is involved in cholesterol transfer and steroidogenesis, a change in its gene expression can result in hyperandrogenism, which is one of the most common symptoms of PCOS.^22^ In rats with EV‐induced PCOS, Azin et al found an increase in *StAR* gene expression.^23^ Hypothyroidism is linked to a decrease in *StAR* gene expression, according to certain studies.^24^ In hypothyroid rats, the loss in testosterone noted earlier is linked to a decrease in *StAR* gene expression.^25^ Current research also shows that the PCOS group had a lower level of *StAR* gene expression than the control group and groups that received 2 and 4 mg/kg of PTU compared with the PCOS group. In a dose‐dependent manner, it was also discovered that decrease was higher in the group that received the most PTU. There was a positive correlation between *StAR* gene expression and testosterone levels. It makes sense considering the importance of the *StAR* gene in steroidogenesis.^26^

Thyroid disease is a chronic inflammatory condition that affects the thyroid gland. B and T lymphocytes play a role in its pathogenesis through the synthesis of thyroglobulin antibody and thyroid peroxidase (TPO) enzyme, as well as a high level of ROS under the influence of the environment or thyroid dysfunction.^27^ Oxidative stress may cause ovarian tissue alterations that are harmful.^28^, ^29^, ^30^ MDA^31^ levels as oxidant increase in thyroid autoimmunity, but SOD^27^ levels as antioxidant decrease, which is similar to the findings of this study. The current results additionally demonstrate that in the group that received 4 mg/kg of PTU, oxidants increased while antioxidants dropped. It suggests that hypothyroidism followed by PCOS can increase the oxidant/antioxidant ratio, causing tissue damage. These findings are consistent with previous studies and confirm the possibility of hypothyroidism as a result of PCOS. As a result, consuming natural antioxidants in the food and vitamin supplements containing antioxidants may be effective strategies for reducing the negative effects of elevated oxidant levels in the simultaneous presence of PCOS and hypothyroidism.

This study is the first to show a link between hypothyroidism and PCOS by looking at the testosterone hormone and the key gene of StAR, as well as oxidants and antioxidants. This could explain the process behind weight gain caused by hypothyroidism and PCOS. Due to the lack of time and cost of measuring the index of inflammatory pathways, other important hormones and genes involved in the subject were not possible, which we intend to complete in future studies.

## AUTHOR CONTRIBUTIONS

**Sara Khodabandeh:** Data curation (equal); formal analysis (equal); investigation (equal); methodology (equal); writing – original draft (equal); writing – review and editing (equal). **Abdolkarim Hosseini:** Conceptualization (equal); data curation (equal); formal analysis (equal); methodology (equal); project administration (equal); software (equal); supervision (equal); validation (equal); visualization (equal); writing – original draft (equal); writing – review and editing (equal). **Homayoun Khazali:** Conceptualization (equal); funding acquisition (equal); methodology (equal); project administration (equal); resources (equal); supervision (equal); validation (equal); visualization (equal); writing – original draft (equal); writing – review and editing (equal). **Vahid Azizi:** Conceptualization (equal); data curation (equal); funding acquisition (equal); project administration (equal); resources (equal); supervision (equal); validation (equal); visualization (equal); writing – original draft (equal); writing – review and editing (equal).

## CONFLICT OF INTEREST

The authors declare that they have no conflict of interest.

## ETHICS STATEMENT

The research design was confirmed from Regional Research Ethics Committees of Shahid Beheshti University, Tehran, Iran (Approval ID: IR.SBU.REC.1400.061).

## Data Availability

The data that support the findings of this study are available from the corresponding author upon reasonable request.
